# Therapeutic Efficacy of Stem Cells Transplantation in Diabetes: Role of Heme Oxygenase

**DOI:** 10.3389/fcell.2016.00080

**Published:** 2016-08-05

**Authors:** Marco Raffaele, Giovanni Li Volti, Ignazio A. Barbagallo, Luca Vanella

**Affiliations:** ^1^Department of Drug Science, University of CataniaCatania, Italy; ^2^Department Biomedical and Biotechnological Science, University of CataniaCatania, Italy

**Keywords:** heme oxygenase, stem cell, diabetes, transplantation, regenerative medicine

## Abstract

The growing data obtained from *in vivo* studies and clinical trials demonstrated the benefit of adult stem cells transplantation in diabetes; although an important limit is represented by their survival after the transplant. To this regard, recent reports suggest that genetic manipulation of stem cells prior to transplantation can lead to enhanced survival and better engraftment. The following review proposes to stimulate interest in the role of heme oxygenase-1 over-expression on transplantation of stem cells in diabetes, focusing on the clinical potential of heme oxygenase protein and activity to restore tissue damage and/or to improve the immunomodulatory properties of transplanted stem cells.

## Diabetes and related complications

Diabetes mellitus includes a series of metabolic disorders due to the lack or diminished effectiveness of insulin secretion (Chiang et al., 2014; American Diabetes Association, 2016). Diabetes can be classified based on its pathogenesis in two distinct classes: the type 1 diabetes, also known as juvenile diabetes, and type 2 diabetes, also known as adult-type diabetes. Both types of diabetes are preceded by a period during which it is observed an impaired glucose homeostasis (Roden, 2016). Prediabetes is an asymptomatic medical condition that does not cause any functional impairment; patients who are in this situation may evolve toward diabetes in varying degrees on the basis of genetic and environmental factors. Prediabetic patients are at an increased risk of developing Type 2 Diabetes Mellitus (Allende-Vigo, 2015). The World Health Organization estimated that 9% of adults worldwide suffer from diabetes, and the total number is predicted to increase by over 50% over the next 20 years (Fonseca, 2009). The epidemiology of diabetes includes population growth, urbanization, aging, ethnicity, increased prevalence of obesity, hypertension, and increased sedentary (Krijnen et al., 2009). Dysfunction of beta cell lead to altered insulin production and secretion resulting in hyperglycemia and glucose intolerance manifesting to diabetes mellitus. Diabetes is associated with premature death, coronary artery disease, and myocardial infarction, stroke, peripheral vascular disease and limb amputation, blindness, kidney failure, and the requirement for dialysis, as well as in several infrequently occurring complications. Diabetic complications are classified into two main groups: the acute and the chronic complications. The former includes diabetic keto-acidosis which is generally encountered in type 1 and nonketotic hyperosmolar syndrome which is common in type 2 diabetes mellitus. The other types of diabetic complications refer to the chronic ones, which may involve several organs and representing the major cause of morbidity and mortality in patients with diabetes mellitus (Tripathi and Srivastava, 2006). The chronic complications of diabetes have been classified as either vascular or non-vascular. The vascular diabetic complications are sub-classified into microvascular including damage to kidneys, eyes, and nerves. The latter leads to diabetic foot ulcer developing into gangrene and amputation. Diabetic ischemic ulcer is an intractable diabetic complication. Recently, transplantation of stem cells is considered as a possible new therapeutic strategy for the treatment of diabetic ulcers. Macrovascular complications are due to damage of the larger blood vessels through a process known as “atherosclerosis,” leading to a narrowing of the vessel lumen and consequent reduction of blood supply in the serving district. Non-vascular complications include gastroporesis and diarrhea, uropathy, or sexual dysfunction, and skin manifestations, gum infection, and complications of eye like cataract and glaucoma (Tripathi and Srivastava, 2006; Cade, 2008). The treatment of diabetes mellitus includes diet (Li Volti et al., 2011; Marrazzo et al., 2014), therapy with insulin and/or oral hypoglycemic agents and pancreas transplantation or pancreatic islets (Logdberg et al., 2003). However, because of the limited availability of donor organs, recent studies have focused on the possibility of using stem cells for the treatment of DM (Li and Ikehara, 2013).

## Health impact of adult stem cells

Multipotent stem cells, which have gradually turned out to play a key role in regenerative medicine therapies, can be retrieved from adult tissue, and bone marrow (BM) represents an important source for these cells. The stem cells derived from BM, as well as umbilical cord blood or the placenta, are all indicated as adult stem cells. The BM mainly contains two types of stem cells: hematopoietic stem cells (HSCs) and mesenchymal stem cells (MSCs). MSCs have been described for the first time by Friedenstein et al. as very similar to fibroblast cells capable of differentiating into cells of the bone tissue (Friedenstein et al., 1968; Hematti, 2008). The fate of a stem cell is determined by its niche, or local micro-environment. Stem cells actively contribute to their environment by secreting cytokines, growth factors and extracellular matrix molecules that act paracrinally and autocrinally. MSCs are considered a critical component of the bone marrow micro-environment, and directly contribute to the development of hematopoietic stem cells, readily distinguishable from MSCs by their cell surface markers. MSCs are considered multipotent stem cells that are capable of self-renewing and differentiating into different functional cell types. The ease of isolation, the high migratory capacity, the relatively high expansion rates, and the ability to avoid the allogeneic responses after transplantation (Sekiya et al., 2002; Chen et al., 2004; Fouillard et al., 2007), make them attractive candidates in regenerative medicine (Pacini, 2014). Recent findings show that MSCs participate in tissue repair processes through various mechanisms including the migration into the damaged tissue (Sordi et al., 2005) and the release of paracrine factors such as cytokines and other trophic factors (Caplan and Dennis, 2006; Salomone et al., 2013).

To this regard, recent animal studies and clinical trials have shown that MSCs are effective in acute myocardial infarction and chronic heart failure and this effect is related to the release of paracrine signals involved in the differentiation of cardiomyocytes, smooth muscle cell, vascular endothelial cells, and stimulating endogenous repair (Boyle et al., 2010). Improvements in myocardial function following stem cells transplantation have been attributed to stem cells differentiation into cardiomyocytes within host myocardium. However, it has been demonstrated that few exogenous stem cells actually engraft and differentiate into cardiomyocyte lineage. Several studies showed that BM-MSCs secrete cytoprotective molecules that reduce apoptosis and necrosis (Baraniak and McDevitt, 2010). Furthermore, it has been demonstrated that resident cardiac progenitor cells are abundantly present within the myocardium in niches preferentially located in the atria and apex and in the ventricle and effectively preserve the integrity of the tissue. Cardiac progenitor cells appear to migrate and accumulate within ischemic and scarred myocardium in order to evoke cardiac regeneration. It has been shown that MSCs can secrete a number of chemotactic factors that may contribute to the activation and migration of cardiac progenitor cells toward areas of injury. Although, it is widely accepted that MSCs functionality is highly affected by sex, age, disease, and the pharmacological treatment of donors, controversies about influence of tissue origins are still debated. These differences may be related to the influence of a modified local environment (niche) present in a different site of the body.

The local microenvironment protects the stem cells and modify their biological behavior. In that regard, the identification and characterization of the stem cells niches has been particularly complex from the time that stem cells are poorly present, and in some cases are devoid of cellular markers that can allow their secure identification. Recently studies described that in the bone marrow, besides MSCs and HSCs, there are different progenitor cells including the endothelial progenitor cells (EPCs) and the mesodermal progenitor cells (MPCs). Pacini at al. isolated MPCs from culture of human bone marrow-derived cells and observed that MPCs differ from MSCs in terms of their morphology and in terms of their quiescent status (Pacini et al., 2010). MPCs have been proven to be capable of generating CFU-F and of differentiating into mesodermal lineages (adipocytes, chondrocytes, and osteoblasts), in the presence of appropriate stimuli, (Pittenger et al., 1999) and through the commitment into an intermediate cell population which is considered “early MSCs.” Furthermore, MPCs may differentiate into endothelial cells when cultured in an appropriate VEGF-containing medium similar to those used in the differentiation of embryonic cells (Trombi et al., 2009). Circulating EPCs are regarded as the cells expressing both stem cell markers and endothelial cell markers. EPCs circulate in the blood and appear to home preferentially to sites of vascular or tissue injury, contributing significantly to both reendothelialization and angiogenesis. Many evidences suggest that BM-derived EPCs have the potential to promote angiogenesis in the postnatal period, thus providing a good rationale for their use in clinical practice for the treatment of cardiovascular diseases. It has been reported that transplantation of EPCs derived from healthy human peripheral blood, significantly increased vascularization, and improved the survival rate after acute liver injury in mice (Taniguchi et al., 2006). Thus, therapeutic approaches using culture-expanded adult stem cells, including progenitor stem cells, could successfully promote regeneration of damaged tissues in cardiovascular diseases.

In addition to these properties, that support the use of stem cells in regenerative medicine, it must be added the capability of this cell type to modulate the T cell response and/or to provide a microenvironment with immunosuppression capacity. The latter ability of MSCs has generated considerable interest in the scientific community since this specific feature could be exploited in the downregulation of the immune response that occurs in graft-vs.-host-disease and autoimmune diseases such as multiple sclerosis, type 1 diabetes and rheumatoid arthritis (Glenn and Whartenby, 2014). During an immune response, MSCs are known to communicate with the inflammatory micro-environment. MSCs express a large number of surface molecules including members of the integrin family and adhesion molecules that promote cellular interactions via receptors binding on immune cells (Najar et al., 2016).

The broad applicability of MSCs in a plethora of metabolic and degenerative disease models is subsequent partially to the ability of MSCs to modulate several cellular component of the innate and adaptive immune system. These features have attracted significant interest in the field of solid organ transplants and some studies have been done to evaluate the effect of the infusion of MSCs simultaneously to organ transplantation in patients treated with standard immunosuppression protocol. MSCs cotransplantation prolonged islet graft survival in all recipients when they also were treated with CTLA4Ig and anti-CD40L (Takahashi et al., 2014). In NOD mice, the administration of a single infusion of MSCs was able to prevent the onset of type 1 diabetes and to retard its progression by inhibiting the accumulation of effector T-cells (Madec et al., 2009). The immunosuppressive effects of MSCs are induced by the activation of several key enzymes, such as nitric oxide synthase, cyclo oxygenase-2, and Heme Oxygenase (HO)-1 (Hinden et al., 2015). Interestingly, human MSCs express HO-1 levels, but HO-1 inhibition significantly reduces the suppressive effects of MSCs, highlighting the key role of HO-1 in the immunosuppression mediated by human MSCs (Chabannes et al., 2007).

## Biological functions of heme oxygenase

HO exists in two forms, HO-1, the inducible form, and HO-2, the constitutive form. Both isoforms degrade heme into biliverdin with the concurrent release of carbon monoxide (CO) and iron (Abraham et al., 2016). In mammals, biliverdin is then reduced by biliverdin reductase to bilirubin (Kapitulnik and Maines, 2009). Iron, bilirubin, and CO, the three byproducts of the HO reaction, possess important biological functions. Both bilirubin and biliverdin have good antioxidant activity and may exert protective effects both *in vivo* and *in vitro* under various experimental conditions (Stocker et al., 1987; Sacerdoti et al., 2005). CO may serve as a second messenger in the central nervous system (Verma et al., 1993), it inhibits endothelial cell apoptosis through the activation of p38MAPK (Otterbein and Choi, 2000; Sacerdoti et al., 2005) and acts as a vasodilator through the stimulation of guanylate cyclase. HO-1 can be induced by many drugs and chemical agents, including statins, aspirin, prostaglandins, eicosanoids, and metals (Marrazzo et al., 2011; Tibullo et al., 2013; Abraham et al., 2016). Furthermore, several natural antioxidant compounds contained in foods and plants have been demonstrated to regulate HO-1 levels in various cellular models (Acquaviva et al., 2009; Vanella et al., 2013, 2016). HO-2 contributes to basal physiological functions while HO-1 represents the major cytoprotective moiety of the HO system, by scavenging ROS and preventing apoptosis (Novo et al., 2011; Burgess et al., 2012; Tibullo et al., 2013). HO may represent a beneficial target to limit the pathogenesis of obesity, diabetes and their complications. It is noteworthy that, an excessive increase in visceral fat is associated with insulin resistance, diabetes and hypertension. Induction of HO-1 in diabetes has been reported to restore the functionality of several mitochondrial carriers, increase Akt phosphorylation and to improve renal function (Di Noia et al., 2006). In addition, HO-1 upregulation leads to a decrease in ROS and LDL levels in many diabetes models. Targeting HO-1 or the products of heme degradation stems from the finding that over-expression of HO-1 increases insulin sensitivity, decreases body weight, and reduces proinflammatory adipokines including TNF-α, IL-6, and MCP-1 (Marino et al., 2012). A decrease in heme levels, consequent to HO-1 activation, limits heme availability for the maturation of gp91phox subunit and assembly of the functional NADPH oxidase which represents the major source of the superoxide anion (Taille et al., 2004).

## Therapeutic potential of HO on stem cells transplantation

MSCs could be exploited for the delivery of specific genes for therapeutic purposes. In this regard, some studies suggest that genetic modification of MSCs prior to transplantation can lead to increased survival, improved engraftment and improved cardiac outcome in models of myocardial infarction (Gnecchi et al., 2006; Wang et al., 2016). Cai at al. demonstrated that pretreatment of human cardiac stem cells with cobalt protoporphyrin (CoPP), an HO-1 inducer, enhanced cells survival, and resulted in great improvement in left ventricular remodeling and in indices of cardiac function after infarction (Cai et al., 2015). In addition to that, previous studies suggested that CoPP increases the survival of cardiomyocytes and restores contractility to adult cardiomyocytes grafts implanted *in vivo* as well as protects the heart from ischemic damage in both normal and diabetes rats (Cao et al., 2012). The use of stem cells as a potential approach to treat diabetes may also offer the possibility to selectively deliver the expression of HO-1 in a cell and organ specific manner without the need of a viral vector or specific promoters. In fact, HO-1 induction may be easily achieved by pharmacological means prior stem cell transplantation in the patients by several agents. In particular, various chemical compounds have been used both *in vivo* and *in vitro* to induce HO-1 such as SnCl_2_ or CoPP. However, there could be some limitations on the possible use of these compounds into a clinical setting because of the metal toxicity. Noteworthy, previous reports showed that natural polyphenols may be used as good inducers of HO-1 because of their ability of activating the Nrf2/Keap1 pathway and may represent a good and safe strategy to induce HO-1 prior to stem cell transplant (Scapagnini et al., 2002; Marrazzo et al., 2011; Barbagallo et al., 2013).

Despite the widespread use of hypoglycemic agents, morbidity and mortality caused by type 1 diabetes mellitus represent a significant burden for society, both in terms of human suffering and cost (Logdberg et al., 2003). Transplantation of pancreatic islets is an important approach to the treatment of diabetes type 1, but after the transplant procedure, cells frequently undergo apoptosis causing a dysfunction of the islets. It has been demonstrated, *in vivo*, that treatment of the mouse donor with carbon monoxide, a reaction product of HO-1, suppresses the proinflammatory response, characterized by an increase of TNF-α, IL-1β, and MCP-1, in the islets after transplantation (Wang et al., 2005). Ikehara's group described the use of stem cells for the treatment of both type 1 and type 2 diabetes (Ikehara, 2003) by a reduction in the development of hyperglycemia and hyperinsulinemia in diabetic mice (Than et al., 1992; Abraham et al., 2008). Chronic hyperglycemia leads to a reduction of the expression of HO-1 and the total enzyme activity, resulting in increased levels of superoxide anion, and cell death (Abraham et al., 2003). Oxidative stress has been shown to play a key role in the pathogenesis of insulin resistance in type 2 diabetes and of cardiovascular complications (Wellen and Hotamisligil, 2005; Ruotsalainen et al., 2008). Previous report demonstrated that the transplantation of bone marrow mesenchymal stem cells (BMMSCs) via intra bone marrow-bone marrow transplantation (IBM-BMT) in conjunction with the induction of HO-1 ameliorate type 2 diabetes mellitus (Li and Ikehara, 2013).

**Figure 1:**
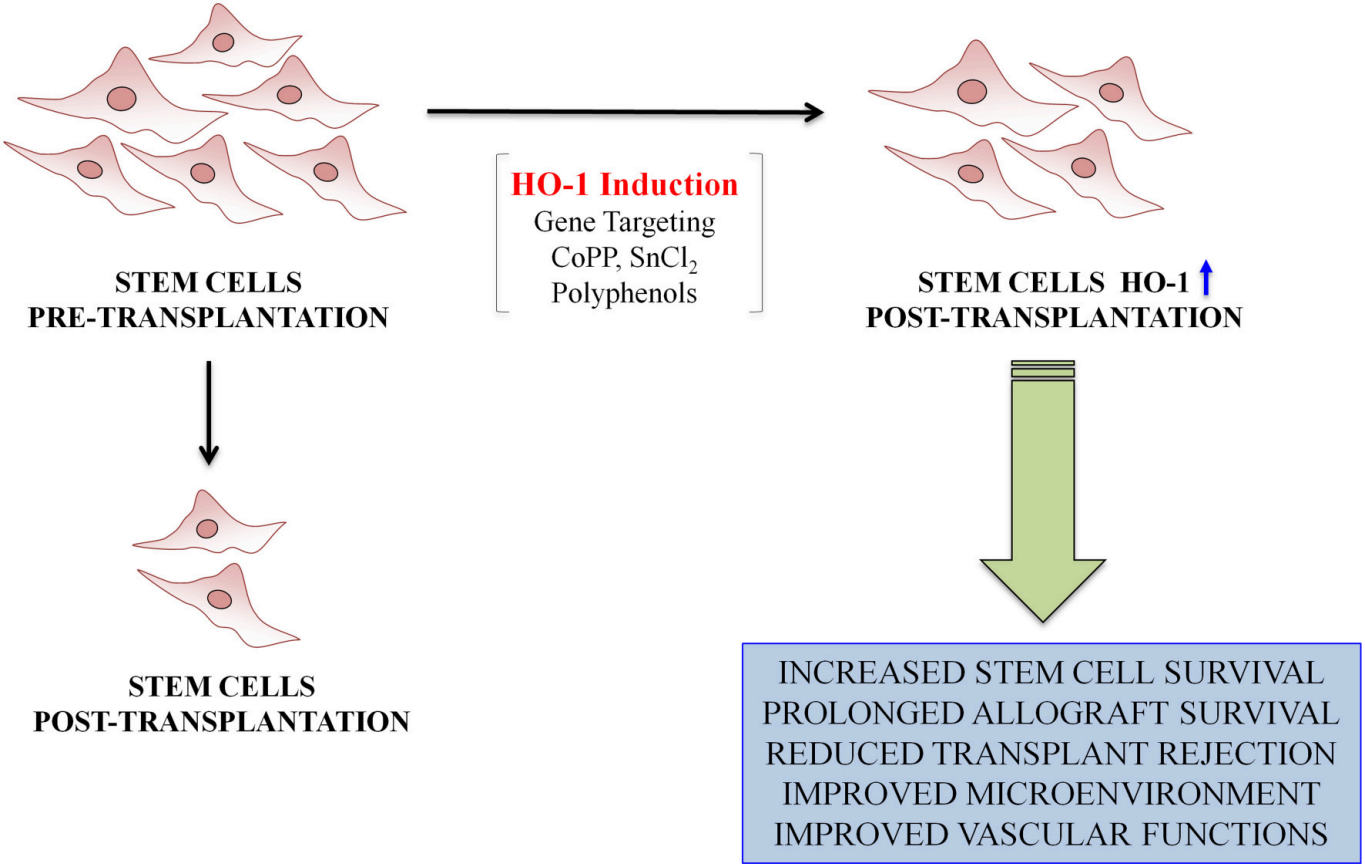
**Schematic representation demonstrating the role of HO-1 in stem cells transplantation**.

Previous studies showed that transplantation of bone marrow stem cells, including MSCs, but not limited to CD34^+^ stem cells, into type 2 diabetic mice restored insulin sensitivity and improved glucose tolerance (Abraham et al., 2008). Pretreatment with a HO-1 inducer followed by the IBM-BMT offers great improvement in diabetes, glucose tolerance, and oxidative stress. The mechanism by which HO-1 improves the effectiveness of IBM-BMT is due to a decrease in superoxide levels, which causes impairment in MSCs function and release of crucial cytokines. Hyperglycemia-induced reactive oxygen species accumulation has been postulated to be a central mediator of diabetes mellitus–induced EPCs dysfunction (Sambuceti et al., 2009). Several studies demonstrated that EPCs represent an important contributor to neovascularization, through the secretion of paracrine angiogenic factors (Urbich et al., 2005). Reduced number and function of EPCs are causally associated with diabetes mellitus–induced impairment in vasculogenesis (Tepper et al., 2002; Sorrentino et al., 2007). A diminished HO-1/AMPK signaling cascade in EPCs may account in part for impaired reendothelialization in diabetes mellitus (Li et al., 2012). EPCs isolated from diabetic mice and transplanted into injured arteries of recipient diabetic mice, didn't show a significant efficacy, whereas the attachment capacity of EPCs isolated from diabetic transgenic mice, over expressing AMPK-HO-1 levels, was comparable to that of EPCs from healthy mice (Li et al., 2012). Treatment of transgenic diabetic mice with a specific HO-1 inhibitor (ZnPPIX) abolished the HO-1 mediated effects, providing a direct evidence that HO-1 upregulation restores the function of damaged EPCs (Li et al., 2012). Experiments *in vitro* revealed that HO-1 is necessary for proper activity of BM-derived stem cells (BMDCs). Indeed, HO-1-deficient BMDCs are less viable when exposed to oxidants and display a weaker proliferation and migration capacity (Grochot-Przeczek et al., 2014). Similar effects were observed in murine mature or progenitor endothelial cells (Jozkowicz et al., 2003; Deshane et al., 2007) and in human endothelium with less active variants of Hmox1 promoter (Taha et al., 2010). In particular, it has been also demonstrated a weaker angiogenic potency of HO-1 −/− BMDCs in capillary sprouting assay, confirming the results obtained earlier in human endothelial cells treated with HO-1 inhibitors or in murine endothelial cells isolated from HO-1 +/+ or HO-1 −/− mice (Grochot-Przeczek et al., 2014). People with diabetes may be at increased risk of developing acute renal failure. Furthermore, numerous studies highlighted diabetes as a major risk factor for the development of acute renal injury (AKI) in hospitalized patients (Johnson et al., 2015). Several studies have shown that the BMSCs transplantation may play a beneficial role following renal ischemic injury, possibly by the paracrine/autocrine mechanisms, or by trans-differentiation into the local cell types (Morigi et al., 2004; Qian et al., 2008; Li et al., 2010). However, after transplantation into the dysfunctional kidney, BMSCs face a pro-oxidant environment characterized by hypoxia, oxidative stress, and inflammation (Mias et al., 2008) that can lead to reduced cell survival and consequently to a decreased therapeutic effect. The oxidative stress leads to stress-induced premature senescence, cell death, and apoptosis of the transplanted BMSCs. Recently, it was reported that over-expression of HO-1, by gene transfection, in BMSCs, was able to improve HO-1-BMSCs survival in the I/R-AKI micro-environment and decrease the levels of MCP-1, TNF-a, and IL-1, leading to a greater improvement of renal function compared to BMSCs treatment alone (Liu et al., 2015). Recently, stem cell transplantation has been considered as a new therapeutic strategy for diabetic foot ulcers. Hou's group demonstrated that the over-expression of HO-1 in BMSCs promoted angiogenesis and wound healing in diabetic ischemic ulcers (Hou et al., 2013). In conclusion, these recent studies demonstrate that induction of HO-1 plays a key role in stem cell survival during transplantation of adult stem cells (Figure 1). Thus, HO-1 represents a new target for designing new compounds with clinical application in the treatment of diabetes.

## Author contributions

MR, GL, IB, LV contributed writing the manuscript, MR searched the scientific literature.

### Conflict of interest statement

The authors declare that the research was conducted in the absence of any commercial or financial relationships that could be construed as a potential conflict of interest.

## References

[B1] Abraham N. G. Junge J. M. Drummond G. S. (2016). Translational significance of heme oxygenase in obesity and metabolic syndrome. Trends Pharmacol. Sci. 37, 17–36. 10.1016/j.tips.2015.09.003 26515032 PMC5488647

[B2] Abraham N. G. Kushida T. McClung J. Weiss M. Quan S. Lafaro R.. (2003). Heme oxygenase-1 attenuates glucose-mediated cell growth arrest and apoptosis in human microvessel endothelial cells. Circ. Res. 93, 507–514. 10.1161/01.RES.0000091828.36599.34 12933701

[B3] Abraham N. G. Li M. Vanella L. Peterson S. J. Ikehara S. Asprinio D. (2008). Bone marrow stem cell transplant into intra-bone cavity prevents type 2 diabetes: role of heme oxygenase-adiponectin. J. Autoimmun. 30, 128–135. 10.1016/j.jaut.2007.12.005 18243659

[B4] Acquaviva R. Lanteri R. Li Destri G. Caltabiano R. Vanella L. Lanzafame S.. (2009). Beneficial effects of rutin and L-arginine coadministration in a rat model of liver ischemia-reperfusion injury. Am. J. Physiol. Gastrointest. Liver Physiol. 296, G664–G670. 10.1152/ajpgi.90609.2008 19109403

[B5] Allende-Vigo M. Z. (2015). Diabetes mellitus prevention. Am. J. Ther. 22, 68–72. 10.1097/MJT.0b013e3182211bae 22020084

[B6] American Diabetes Association (2016). 2 Classification and diagnosis of diabetes. Diabetes Care 39(Suppl. 1), S13–S22. 10.2337/dc16-S005 26696675

[B7] Baraniak P. R. McDevitt T. C. (2010). Stem cell paracrine actions and tissue regeneration. Regen. Med. 5, 121–143. 10.2217/rme.09.74 20017699 PMC2833273

[B8] Barbagallo I. Galvano F. Frigiola A. Cappello F. Riccioni G. Murabito P.. (2013). Potential therapeutic effects of natural heme oxygenase-1 inducers in cardiovascular diseases. Antioxid. Redox Signal. 18, 507–521. 10.1089/ars.2011.4360 23025298

[B9] Boyle A. J. McNiece I. K. Hare J. M. (2010). Mesenchymal stem cell therapy for cardiac repair. Methods Mol. Biol. 660, 65–84. 10.1007/978-1-60761-705-1_5 20680813

[B10] Burgess A. P. Vanella L. Bellner L. Gotlinger K. Falck J. R. Abraham N. G.. (2012). Heme oxygenase (HO-1) rescue of adipocyte dysfunction in HO-2 deficient mice via recruitment of epoxyeicosatrienoic acids (EETs) and adiponectin. Cell. Physiol. Biochem. 29, 99–110. 10.1159/000337591 22415079 PMC3711769

[B11] Cade W. T. (2008). Diabetes-related microvascular and macrovascular diseases in the physical therapy setting. Phys. Ther. 88, 1322–1335. 10.2522/ptj.20080008 18801863 PMC2579903

[B12] Cai C. Guo Y. Teng L. Nong Y. Tan M. Book M. J.. (2015). Preconditioning human cardiac stem cells with an HO-1 inducer exerts beneficial effects after cell transplantation in the infarcted murine heart. Stem Cells 33, 3596–3607. 10.1002/stem.2198 26299779 PMC4766973

[B13] Cao J. Vecoli C. Neglia D. Tavazzi B. Lazzarino G. Novelli M.. (2012). Cobalt-protoporphyrin improves heart function by blunting oxidative stress and restoring no synthase equilibrium in an animal model of experimental diabetes. Front. Physiol. 3:160. 10.3389/fphys.2012.00160 22675305 PMC3366474

[B14] Caplan A. I. Dennis J. E. (2006). Mesenchymal stem cells as trophic mediators. J. Cell. Biochem. 98, 1076–1084. 10.1002/jcb.20886 16619257

[B15] Chabannes D. Hill M. Merieau E. Rossignol J. Brion R. Soulillou J. P.. (2007). A role for heme oxygenase-1 in the immunosuppressive effect of adult rat and human mesenchymal stem cells. Blood 110, 3691–3694. 10.1182/blood-2007-02-075481 17684157

[B16] Chen S. L. Fang W. W. Ye F. Liu Y. H. Qian J. Shan S. J.. (2004). Effect on left ventricular function of intracoronary transplantation of autologous bone marrow mesenchymal stem cell in patients with acute myocardial infarction. Am. J. Cardiol. 94, 92–95. 10.1016/j.amjcard.2004.03.034 15219514

[B17] Chiang J. L. Kirkman M. S. Laffel L. M. Peters A. L. (2014). Type 1 Diabetes Sourcebook A: type 1 diabetes through the life span: a position statement of the American Diabetes Association. Diabetes Care 37, 2034–2054. 10.2337/dc14-1140 24935775 PMC5865481

[B18] Deshane J. Chen S. Caballero S. Grochot-Przeczek A. Was H. Li Calzi S.. (2007). Stromal cell-derived factor 1 promotes angiogenesis via a heme oxygenase 1-dependent mechanism. J. Exp. Med. 204, 605–618. 10.1084/jem.20061609 17339405 PMC1855437

[B19] Di Noia M. A. Van Driesche S. Palmieri F. Yang L. M. Quan S. Goodman A. I.. (2006). Heme oxygenase-1 enhances renal mitochondrial transport carriers and cytochrome C oxidase activity in experimental diabetes. J. Biol. Chem. 281, 15687–15693. 10.1074/jbc.M510595200 16595661

[B20] Fonseca V. A. (2009). Defining and characterizing the progression of type 2 diabetes. Diabetes Care 32(Suppl. 2), S151–S156. 10.2337/dc09-S301 19875543 PMC2811457

[B21] Fouillard L. Chapel A. Bories D. Bouchet S. Costa J. M. Rouard H.. (2007). Infusion of allogeneic-related HLA mismatched mesenchymal stem cells for the treatment of incomplete engraftment following autologous haematopoietic stem cell transplantation. Leukemia 21, 568–570. 10.1038/sj.leu.2404550 17252011

[B22] Friedenstein A. J. Petrakova K. V. Kurolesova A. I. Frolova G. P. (1968). Heterotopic of bone marrow. analysis of precursor cells for osteogenic and hematopoietic tissues. Transplantation 6, 230–247. 10.1097/00007890-196803000-00009 5654088

[B23] Glenn J. D. Whartenby K. A. (2014). Mesenchymal stem cells: emerging mechanisms of immunomodulation and therapy. World J. Stem Cells 6, 526–539. 10.4252/wjsc.v6.i5.526 25426250 PMC4178253

[B24] Gnecchi M. He H. Noiseux N. Liang O. D. Zhang L. Morello F.. (2006). Evidence supporting paracrine hypothesis for Akt-modified mesenchymal stem cell-mediated cardiac protection and functional improvement. FASEB J. 20, 661–669. 10.1096/fj.05-5211com 16581974

[B25] Grochot-Przeczek A. Kotlinowski J. Kozakowska M. Starowicz K. Jagodzinska J. Stachurska A.. (2014). Heme oxygenase-1 is required for angiogenic function of bone marrow-derived progenitor cells: role in therapeutic revascularization. Antioxid. Redox Signal. 20, 1677–1692. 10.1089/ars.2013.5426 24206054 PMC3961799

[B26] Hematti P. (2008). Role of mesenchymal stromal cells in solid organ transplantation. Transplant. Rev. 22, 262–273. 10.1016/j.trre.2008.05.002 18656340 PMC2576746

[B27] Hinden L. Shainer R. Almogi-Hazan O. Or R. (2015). *Ex vivo* induced regulatory human/murine mesenchymal stem cells as immune modulators. Stem Cells 33, 2256–2267. 10.1002/stem.2026 25850816

[B28] Hou C. Shen L. Huang Q. Mi J. Wu Y. Yang M.. (2013). The effect of heme oxygenase-1 complexed with collagen on MSC performance in the treatment of diabetic ischemic ulcer. Biomaterials 34, 112–120. 10.1016/j.biomaterials.2012.09.022 23059006

[B29] Ikehara S. (2003). A novel strategy for allogeneic stem cell transplantation: perfusion method plus intra-bone marrow injection of stem cells. Exp. Hematol. 31, 1142–1146. 10.1016/j.exphem.2003.08.020 14662319

[B30] Johnson F. Phillips D. Talabani B. Wonnacott A. Meran S. Phillips A. O. (2015). The impact of acute kidney injury in diabetes mellitus. Nephrology 21, 506–511. 10.1111/nep.12649 26452246

[B31] Jozkowicz A. Huk I. Nigisch A. Weigel G. Dietrich W. Motterlini R.. (2003). Heme oxygenase and angiogenic activity of endothelial cells: stimulation by carbon monoxide and inhibition by tin protoporphyrin-IX. Antioxid. Redox Signal. 5, 155–162. 10.1089/152308603764816514 12716475

[B32] Kapitulnik J. Maines M. D. (2009). Pleiotropic functions of biliverdin reductase cellular signaling and generation of cytoprotective and cytotoxic bilirubin. Trends Pharmacol. Sci. 30, 129–137. 10.1016/j.tips.2008.12.003 19217170

[B33] Krijnen P. A. Simsek S. Niessen H. W. (2009). Apoptosis in diabetes. Apoptosis 14, 1387–1388. 10.1007/s10495-009-0419-6 19856207 PMC2773035

[B34] Li F. Y. Lam K. S. Tse H. F. Chen C. Wang Y. Vanhoutte P. M.. (2012). Endothelium-selective activation of AMP-activated protein kinase prevents diabetes mellitus-induced impairment in vascular function and reendothelialization via induction of heme oxygenase-1 in mice. Circulation 126, 1267–1277. 10.1161/CIRCULATIONAHA.112.108159 22851545

[B35] Li K. Han Q. Yan X. Liao L. Zhao R. C. (2010). Not a process of simple vicariousness, the differentiation of human adipose-derived mesenchymal stem cells to renal tubular epithelial cells plays an important role in acute kidney injury repairing. Stem Cells Dev. 19, 1267–1275. 10.1089/scd.2009.0196 19874085

[B36] Li M. Ikehara S. (2013). Bone marrow stem cell as a potential treatment for diabetes. J. Diabetes Res. 2013:329596. 10.1155/2013/329596 23671865 PMC3647566

[B37] Liu N. Wang H. Han G. Tian J. Hu W. Zhang J. (2015). Alleviation of apoptosis of bone marrow-derived mesenchymal stem cells in the acute injured kidney by heme oxygenase-1 gene modification. Int. J. Biochem. Cell Biol. 69, 85–94. 10.1016/j.biocel.2015.10.007 26456668

[B38] Li Volti G. Salomone S. Sorrenti V. Mangiameli A. Urso V. Siarkos I.. (2011). Effect of silibinin on endothelial dysfunction and ADMA levels in obese diabetic mice. Cardiovasc. Diabetol. 10:62. 10.1186/1475-2840-10-62 21756303 PMC3152512

[B39] Logdberg L. Sgan S. L. Larsen C. P. Hillyer C. D. (2003). Islet transplantation, stem cells, and transfusion medicine. Transfus. Med. Rev. 17, 95–109. 10.1053/tmrv.2003.50006 12733103

[B40] Madec A. M. Mallone R. Afonso G. Abou Mrad E. Mesnier A. Eljaafari A.. (2009). Mesenchymal stem cells protect NOD mice from diabetes by inducing regulatory T cells. Diabetologia 52, 1391–1399. 10.1007/s00125-009-1374-z 19421731

[B41] Marino J. S. Peterson S. J. Li M. Vanella L. Sodhi K. Hill J. W.. (2012). ApoA-1 mimetic restores adiponectin expression and insulin sensitivity independent of changes in body weight in female obese mice. Nutr. Diabetes 2, e33. 10.1038/nutd.2012.4 23169576 PMC3341710

[B42] Marrazzo G. Barbagallo I. Galvano F. Malaguarnera M. Gazzolo D. Frigiola A.. (2014). Role of dietary and endogenous antioxidants in diabetes. Crit. Rev. Food Sci. Nutr. 54, 1599–1616. 10.1080/10408398.2011.644874 24580561

[B43] Marrazzo G. Bosco P. La Delia F. Scapagnini G. Di Giacomo C. Malaguarnera M.. (2011). Neuroprotective effect of silibinin in diabetic mice. Neurosci. Lett. 504, 252–256. 10.1016/j.neulet.2011.09.041 21970972

[B44] Mias C. Trouche E. Seguelas M. H. Calcagno F. Dignat-George F. Sabatier F.. (2008). *Ex vivo* pretreatment with melatonin improves survival, proangiogenic/mitogenic activity, and efficiency of mesenchymal stem cells injected into ischemic kidney. Stem Cells 26, 1749–1757. 10.1634/stemcells.2007-1000 18467662

[B45] Morigi M. Imberti B. Zoja C. Corna D. Tomasoni S. Abbate M.. (2004). Mesenchymal stem cells are renotropic, helping to repair the kidney and improve function in acute renal failure. J. Am. Soc. Nephrol. 15, 1794–1804. 10.1097/01.ASN.0000128974.07460.34 15213267

[B46] Najar M. Raicevic G. Fayyad-Kazan H. Bron D. Toungouz M. Lagneaux L. (2016). Mesenchymal stromal cells and immunomodulation: a gathering of regulatory immune cells. Cytotherapy 18, 160–171. 10.1016/j.jcyt.2015.10.011 26794710

[B47] Novo G. Cappello F. Rizzo M. Fazio G. Zambuto S. Tortorici E.. (2011). Hsp60 and heme oxygenase-1 (Hsp32) in acute myocardial infarction. Transl. Res. 157, 285–292. 10.1016/j.trsl.2011.01.003 21497776

[B48] Otterbein L. E. Choi A. M. (2000). Heme oxygenase: colors of defense against cellular stress. Am. J. Physiol. Lung Cell. Mol. Physiol. 279, L1029–L1037. Available online at: ajplung.physiology.org/11076792 10.1152/ajplung.2000.279.6.L1029

[B49] Pacini S. (2014). Deterministic and stochastic approaches in the clinical application of mesenchymal stromal cells (MSCs). Front. Cell Dev. Biol. 2:50. 10.3389/fcell.2014.00050 25364757 PMC4206995

[B50] Pacini S. Carnicelli V. Trombi L. Montali M. Fazzi R. Lazzarini E.. (2010). Constitutive expression of pluripotency-associated genes in mesodermal progenitor cells (MPCs). PLoS ONE 5:e9861. 10.1371/journal.pone.0009861 20360837 PMC2845604

[B51] Pittenger M. F. Mackay A. M. Beck S. C. Jaiswal R. K. Douglas R. Mosca J. D.. (1999). Multilineage potential of adult human mesenchymal stem cells. Science 284, 143–147. 10.1126/science.284.5411.143 10102814

[B52] Qian H. Yang H. Xu W. Yan Y. Chen Q. Zhu W.. (2008). Bone marrow mesenchymal stem cells ameliorate rat acute renal failure by differentiation into renal tubular epithelial-like cells. Int. J. Mol. Med. 22, 325–332. 10.3892/ijmm_00000026 18698491

[B53] Roden M. (2016). Diabetes mellitus: definition, classification and diagnosis. Wien. Klin. Wochenschr. 128(Suppl. 2), 37–40. 10.1007/s00508-015-0931-3 27052219

[B54] Ruotsalainen E. Vauhkonen I. Salmenniemi U. Pihlajamaki J. Punnonen K. Kainulainen S.. (2008). Markers of endothelial dysfunction and low-grade inflammation are associated in the offspring of type 2 diabetic subjects. Atherosclerosis 197, 271–277. 10.1016/j.atherosclerosis.2007.04.021 17560580

[B55] Sacerdoti D. Colombrita C. Ghattas M. H. Ismaeil E. F. Scapagnini G. Bolognesi M.. (2005). Heme oxygenase-1 transduction in endothelial cells causes downregulation of monocyte chemoattractant protein-1 and of genes involved in inflammation and growth. Cell. Mol. Biol. 51, 363–370. 16309586

[B56] Salomone F. Barbagallo I. Puzzo L. Piazza C. Li Volti G. (2013). Efficacy of adipose tissue-mesenchymal stem cell transplantation in rats with acetaminophen liver injury. Stem Cell Res. 11, 1037–1044. 10.1016/j.scr.2013.07.003 23954692

[B57] Sambuceti G. Morbelli S. Vanella L. Kusmic C. Marini C. Massollo M.. (2009). Diabetes impairs the vascular recruitment of normal stem cells by oxidant damage, reversed by increases in pAMPK, heme oxygenase-1, and adiponectin. Stem Cells 27, 399–407. 10.1634/stemcells.2008-0800 19038792 PMC2729677

[B58] Scapagnini G. Foresti R. Calabrese V. Giuffrida Stella A. M. Green C. J. Motterlini R. (2002). Caffeic acid phenethyl ester and curcumin: a novel class of heme oxygenase-1 inducers. Mol. Pharmacol. 61, 554–561. 10.1124/mol.61.3.554 11854435

[B59] Sekiya I. Larson B. L. Smith J. R. Pochampally R. Cui J. G. Prockop D. J. (2002). Expansion of human adult stem cells from bone marrow stroma: conditions that maximize the yields of early progenitors and evaluate their quality. Stem Cells 20, 530–541. 10.1634/stemcells.20-6-530 12456961

[B60] Sordi V. Malosio M. L. Marchesi F. Mercalli A. Melzi R. Giordano T.. (2005). Bone marrow mesenchymal stem cells express a restricted set of functionally active chemokine receptors capable of promoting migration to pancreatic islets. Blood 106, 419–427. 10.1182/blood-2004-09-3507 15784733

[B61] Sorrentino S. A. Bahlmann F. H. Besler C. Muller M. Schulz S. Kirchhoff N.. (2007). Oxidant stress impairs *in vivo* reendothelialization capacity of endothelial progenitor cells from patients with type 2 diabetes mellitus: restoration by the peroxisome proliferator-activated receptor-gamma agonist rosiglitazone. Circulation 116, 163–173. 10.1161/CIRCULATIONAHA.106.684381 17592079

[B62] Stocker R. Yamamoto Y. McDonagh A. F. Glazer A. N. Ames B. N. (1987). Bilirubin is an antioxidant of possible physiological importance. Science 235, 1043–1046. 10.1126/science.3029864 3029864

[B63] Taha H. Skrzypek K. Guevara I. Nigisch A. Mustafa S. Grochot-Przeczek A.. (2010). Role of heme oxygenase-1 in human endothelial cells: lesson from the promoter allelic variants. Arterioscler. Thromb. Vasc. Biol. 30, 1634–1641. 10.1161/ATVBAHA.110.207316 20508205 PMC2906705

[B64] Taille C. El-Benna J. Lanone S. Dang M. C. Ogier-Denis E. Aubier M.. (2004). Induction of heme oxygenase-1 inhibits NAD(P)H oxidase activity by down-regulating cytochrome b558 expression via the reduction of heme availability. J. Biol. Chem. 279, 28681–28688. 10.1074/jbc.M310661200 15123630

[B65] Takahashi T. Tibell A. Ljung K. Saito Y. Gronlund A. Osterholm C.. (2014). Multipotent mesenchymal stromal cells synergize with costimulation blockade in the inhibition of immune responses and the induction of Foxp3+ regulatory T cells. Stem Cells Transl. Med. 3, 1484–1494. 10.5966/sctm.2014-0012 25313200 PMC4250203

[B66] Taniguchi E. Kin M. Torimura T. Nakamura T. Kumemura H. Hanada S.. (2006). Endothelial progenitor cell transplantation improves the survival following liver injury in mice. Gastroenterology 130, 521–531. 10.1053/j.gastro.2005.10.050 16472604

[B67] Tepper O. M. Galiano R. D. Capla J. M. Kalka C. Gagne P. J. Jacobowitz G. R.. (2002). Human endothelial progenitor cells from type II diabetics exhibit impaired proliferation, adhesion, and incorporation into vascular structures. Circulation 106, 2781–2786. 10.1161/01.CIR.0000039526.42991.93 12451003

[B68] Than S. Ishida H. Inaba M. Fukuba Y. Seino Y. Adachi M.. (1992). Bone marrow transplantation as a strategy for treatment of non-insulin-dependent diabetes mellitus in KK-Ay mice. J. Exp. Med. 176, 1233–1238. 10.1084/jem.176.4.1233 1402665 PMC2119408

[B69] Tibullo D. Barbagallo I. Giallongo C. La Cava P. Parrinello N. Vanella L.. (2013). Nuclear translocation of heme oxygenase-1 confers resistance to imatinib in chronic myeloid leukemia cells. Curr. Pharm. Des. 19, 2765–2770. 10.2174/1381612811319150012 23092325

[B70] Tripathi B. K. Srivastava A. K. (2006). Diabetes mellitus: complications and therapeutics. Med. Sci. Monit. 12, RA130–RA147. Available online at: www.medscimonit.com/16810145

[B71] Trombi L. Pacini S. Montali M. Fazzi R. Chiellini F. Ikehara S.. (2009). Selective culture of mesodermal progenitor cells. Stem Cells Dev. 18, 1227–1234. 10.1089/scd.2009.0054 19331526

[B72] Urbich C. Aicher A. Heeschen C. Dernbach E. Hofmann W. K. Zeiher A. M.. (2005). Soluble factors released by endothelial progenitor cells promote migration of endothelial cells and cardiac resident progenitor cells. J. Mol. Cell. Cardiol. 39, 733–742. 10.1016/j.yjmcc.2005.07.003 16199052

[B73] Vanella L. Di Giacomo C. Acquaviva R. Barbagallo I. Li Volti G. Cardile V.. (2013). Effects of ellagic Acid on angiogenic factors in prostate cancer cells. Cancers 5, 726–738. 10.3390/cancers5020726 24216999 PMC3730328

[B74] Vanella L. Tibullo D. Godos J. Pluchinotta F. R. Di Giacomo C. Sorrenti V.. (2016). Caffeic acid phenethyl ester regulates PPAR's levels in stem cells-derived adipocytes. PPAR Res. 2016:7359521. 10.1155/2016/7359521 26904104 PMC4745343

[B75] Verma A. Hirsch D. J. Glatt C. E. Ronnett G. V. Snyder S. H. (1993). Carbon monoxide: a putative neural messenger. Science 259, 381–384. 10.1126/science.7678352 7678352

[B76] Wang H. Lee S. S. Gao W. Czismadia E. McDaid J. Ollinger R.. (2005). Donor treatment with carbon monoxide can yield islet allograft survival and tolerance. Diabetes 54, 1400–1406. 10.2337/diabetes.54.5.1400 15855326

[B77] Wang J. Hu X. Jiang H. (2016). MSCs modified with HO-1 gene transplantation: A novel therapeutic approach for attenuating heart failure. Int. J. Cardiol. 214, 159–160. 10.1016/j.ijcard.2016.03.193 27061652

[B78] Wellen K. E. Hotamisligil G. S. (2005). Inflammation, stress, and diabetes. J. Clin. Invest. 115, 1111–1119. 10.1172/JCI200525102 15864338 PMC1087185

